# Retraction: Role of institutions in promoting finance in emerging markets: A panel data analysis

**DOI:** 10.1371/journal.pone.0333749

**Published:** 2025-10-09

**Authors:** The PLOS One Editors

The *PLOS One* Editors retract this article [1] because it was identified as one of a series of submissions for which we have concerns about potential manipulation of the publication process, peer review integrity, and authorship. These concerns call into question the validity and provenance of the reported results. We regret that the issues were not identified prior to the article’s publication.

All authors did not agree with the retraction.

## References

[1] Aman A, Khan MA, Khan MA, Haider A, Oláh J, Fenyves V. Role of institutions in promoting finance in emerging markets: A panel data analysis. PLoS One. 2023;18(3):e0280849. doi: 10.1371/journal.pone.0280849 36952556 PMC10035861

